# Predictors of the development of myocarditis or acute renal failure in patients with leptospirosis: An observational study

**DOI:** 10.1186/1471-2334-12-4

**Published:** 2012-01-13

**Authors:** Dinesh LB Dassanayake, Harith Wimalaratna, Damith Nandadewa, Asanka Nugaliyadda, Champa N Ratnatunga, Suneth B Agampodi

**Affiliations:** 1 Registrar in Medicine, Teaching Hospital Kandy, Kandy, Sri Lanka; 2 Consultant Physician, Teaching Hospital Kandy, Kandy, Sri Lanka; 3 Intern House Officers, Teaching Hospital Kandy, Kandy, Sri Lanka; 4 Lecturer in Community Medicine, Faculty of Medicine and Allied Sciences, University of Rajarata, Mihintale, Anuradhapura, Sri Lanka

**Keywords:** Myocarditis, Acute renal failure, Leptospirosis

## Abstract

**Background:**

Leptospirosis has a varied clinical presentation with complications like myocarditis and acute renal failure. There are many predictors of severity and mortality including clinical and laboratory parameters. Early detection and treatment can reduce complications. Therefore recognizing the early predictors of the complications of leptospirosis is important in patient management. This study was aimed at determining the clinical and laboratory predictors of myocarditis or acute renal failure.

**Methods:**

This was a prospective descriptive study carried out in the Teaching Hospital, Kandy, from 1st July 2007 to 31st July 2008. Patients with clinical features compatible with leptospirosis case definition were confirmed using the Microscopic Agglutination Test (MAT). Clinical features and laboratory measures done on admission were recorded. Patients were observed for the development of acute renal failure or myocarditis. Chi-square statistics, Fisher's exact test and Mann-Whitney *U *test were used to compare patients with and without complications. A logistic regression model was used to select final predictor variables.

**Results:**

Sixty two confirmed leptospirosis patients were included in the study. Seven patients (11.3%) developed acute renal failure and five (8.1%) developed myocarditis while three (4.8%) had both acute renal failure and myocarditis. Conjunctival suffusion - 40 (64.5%), muscle tenderness - 28 (45.1%), oliguria - 20 (32.2%), jaundice - 12 (19.3%), hepatomegaly - 10 (16.1%), arrhythmias (irregular radial pulse) - 8 (12.9%), chest pain - 6 (9.7%), bleeding - 5 (8.1%), and shortness of breath (SOB) 4 (6.4%) were the common clinical features present among the patients. Out of these, only oliguria {odds ratio (OR) = 4.14 and 95% confidence interval (CI) 1.003-17.261}, jaundice (OR = 5.13 and 95% CI 1.149-28.003), and arrhythmias (OR = 5.774 and 95% CI 1.001-34.692), were predictors of myocarditis or acute renal failure and none of the laboratory measures could predict the two complications.

**Conclusions:**

This study shows that out of clinical and laboratory variables, only oliguria, jaundice and arrhythmia are strong predictors of development of acute renal failure or myocarditis in patients with leptospirosis presented to Teaching Hospital of Kandy, Sri Lanka.

## Background

Leptospirosis is endemic in both urban and rural areas of Sri Lanka and there had been many outbreaks in the recent past [1]. In year 2007, 2187 suspected cases were reported in Sri Lanka while 147 cases were reported from the Kandy District [2]. In the most recent epidemic of leptospirosis in 2008, the number of cases have increased to 7099 and the number of deaths has gone up to 204 (6 fold increase compared to 2007) [3].

Clinical leptospirosis can vary from a mild non-specific influenza-like infection to a severe disease, where serious complications like acute renal failure, myocarditis, pulmonary haemorrhage and liver failure are reported [4]. Case fatality rates are varied significantly between 3% and 54% based on the affected organ-system [5,6]. Cardiac and renal involvements are two of the most dreaded complications of leptospirosis. Rajiv et al. has shown that 70% of the patients with serologically proven leptospirosis had electrocardiographic abnormalities, with atrial fibrillation being the commonest major arrhythmia noted [7]. The same study showed that 36% of the patients had conduction system abnormalities and 30% had T wave changes. Another case series has reported atrio - ventricular block in 44% of patients with leptospirosis [8]. A glycoprotein fraction of leptospiral cell wall has been incriminated in the pathogenesis of these rhythm disturbances. Other reported cardiac abnormalities include myocarditis and endocarditis [9].

Renal involvement is the most serious complication in leptospirosis and is the commonest cause of death [10]. Two mechanisms have been postulated in the production of leptospiral renal failure: (1) direct nephrotoxicity brought about by various endotoxins or endotoxin-like substances and (2) anoxic effect due to disturbances in renal circulation. The typical lesion is tubulointerstitial nephritis, characterized by interstitial edema and dense focal infiltration of predominantly mononuclear cells. Tubular changes are degenerative in nature and affect principally the proximal tubules. Intravascular volume depletion causing the vasomotor nephropathy in this disease has been postulated to occur from capillary leakage with associated loss of fluid and protein, hence the proteinuria [10].

Severe leptospirosis may carry a high mortality if treatment is not instituted early. Poor prognostic markers in leptospirosis reported in various studies include hypotension, oliguria and hyperkalemia [11]. Other studies have reported dyspnea, white blood cell count greater than 12,900/mm^3 ^[12], repolarization abnormalities on electrocardiograms, alveolar infiltrates [13], hemoptysis, metabolic acidosis, and thrombocytopenia. In hospitals that have adequate facilities, patients with these risk factors should preferably be treated in an intensive care unit [14].

The beginning of early pertinent antimicrobial therapy within 4-5 days after the onset of illness, proper supportive therapy and use of dialysis to treat renal failure has reduced the leptospirosis-related mortality [15]. Therefore early predictors of complications are helpful in detecting and managing patients with leptospirosis and also to minimize the mortality. The present study was aimed at determining which clinical and laboratory findings can predict the development of myocarditis or acute renal failure.

## Methods

### Study setting

This study was carried out in the medical wards of Teaching Hospital Kandy, Sri Lanka, which is a tertiary care hospital. This medical unit treats about 10,000 patients per year and 10% of the admissions are due to fever, out of which substantial proportion is due to leptospirosis. Ethical approval was granted by the Ethical Committee of the Teaching Hospital {No: AB (II)/ETHICAL/2007}. Study population included patients with leptospirosis who were admitted to wards 33 and 35, from 1st July 2007 to 31st July 2008. Patients without a serological proof or who died before serologically proving leptospirosis were excluded.

### Procedure

Patients with clinical features of leptospirosis were screened using a symptom check list and interviewer administered questionnaire to diagnose leptospirosis according to the leptospirosis case definition. Surveillance case definition for diagnosis of leptospirosis which was published by the Epidemiology Unit was used for the present study [16]. Nine clinical features which were thought to predict complications were recorded. The clinical features were chest pain, shortness of breath (SOB), oliguria, jaundice, bleeding, conjunctival suffusion, arrhythmias (irregular radial pulse), muscle tenderness, and hepatomegaly. Blood samples were taken on admission for Hemoglobin (Hb), packed cell volume (PCV), white blood cell count (WBC), bilirubin, platelet count (PLC), blood urea (BU), serum potassium (K), and creatinine.

Leptospirosis was confirmed using the genus specific Microscopic Agglutination Test (MAT) done on blood samples drawn on 7th day of fever. Informed written consent was obtained from patients before participating in the study as well as before blood sampling. The MAT was done in Medical Research Institute (MRI) Colombo which uses the non pathogenic Patoc strain of *Leptospira biflexa*. A positive MAT test was defined as a titre of ≥1: 800 in acute phase serum. This is the titre specified by the MRI which is the national reference laboratory [17].

Only the laboratory confirmed patients were included in the study. The patients were observed for the development of acute renal failure or myocarditis alone or in combination.

Acute renal failure was defined as the presence of one of the following criteria

• An increase in serum creatinine of > 0.5 mg/dl.

• An increase in serum creatinine of > 50% from baseline.

• A reduction in calculated creatinine clearance of > 50%.

• Need for dialysis.

Myocarditis was defined as reduction in systolic blood pressure below 100 mm Hg with one of the following criteria.

• Presence of transient ST segment changes, T wave changes, or conduction abnormalities in the absence of electrolyte imbalances.

• Reduction of ejection fraction < 40% with characteristic echocardiographic evidence of myocarditis.

• Need for inotropic support.

### Statistical analysis

Descriptive analyses of patients with and without complications were done using proportions and percentages. Chi-square statistics, Fisher's exact test and Mann-Whitney *U *test were used to compare patients with and without complications. All variables with a "*p*" value less than 0.1 were selected for the predictor model. A logistic regression model was used to evaluate the predictor variables. A backward stepwise elimination of variables was carried out to select final variables included in the model. Results were analyzed using SPSS software.

## Results

Sixty two confirmed leptospirosis patients were included in the study. Mean age of the confirmed cases of leptospirosis was 39 years with a standard deviation of 19. The male to female ratio was 3:1. Seven patients (11.3%) developed acute renal failure and five (8.1%) developed myocarditis while three (4.8%) had acute renal failure and myocarditis together. Table 1 shows the distribution of confirmed cases of leptospirosis by clinical features and complications (acute renal failure or myocarditis).

**Table 1:** Distribution of confirmed cases of leptospirosis by clinical features and complications (acute renal failure or myocarditis)

Clinical feature		Complications (acute renal failure or myocarditis)				Significance	
				
		No		Yes			
				
		n	%	n	%		
Oliguria	No	35	83.3	7	16.7	Fisher's	

	Yes	12	60.0	8	40.0	*p*	.060

Jaundice	No	41	82.0	9	18.0	Fisher's	

	Yes	6	50.0	6	50.0	*p*	.054

Bleeding	No	43	75.4	14	24.6	Fisher's	

	Yes	4	80.0	1	20.0	*p*	.672

Conjunctival suffusion	No	18	81.8	4	18.2	Fisher's	

	Yes	29	72.5	11	27.5	*p*	1.000

Arrhythmias	No	44	81.5	10	18.5	Fisher's	

	Yes	3	37.5	5	62.5	*p*	.016*

Chest pain	No	42	75.0	14	25.0	Fisher's	

	Yes	5	83.3	1	16.7	*p*	.549

SOB	No	44	75.9	14	24.1	Fisher's	

	Yes	3	75.0	1	25.0	*p*	.680

Muscle tenderness	No	26	76.5	8	23.5	Chi-square	

	Yes	21	75.0	7	25.0	*p*	.893

Hepatomegaly	No	41	78.8	11	21.2	Fisher's	

	Yes	6	60.0	4	40.0	*p*	.189

*Significant

Of the 8 patients who had arrhythmias, 5 (62.5%) developed complications and among 54 patients without arrhythmias only 10 (18.5%) developed complications. This observed difference was statistically significant.

Table 2 shows the distribution of confirmed cases of leptospirosis by laboratory measures and complications (acute renal failure or myocarditis).

**Table 2:** Distribution of confirmed cases of leptospirosis by laboratory measure and complications (acute renal failure or myocarditis)

Laboratory measure	Complications (acute renal failure or myocarditis)				Significance^1 ^
		
	No		Yes		
		
	Median	IQR	Median	IQR	***p***
Hb (g/L)	12	12-13	11	11-12	.017*

PCV (%)	38	36-42	36	28-37	.041*

WBC (10^9^)	8	6-10	11	10-17	.001*

Bilirubin (mic mol/L)	50	14-80	159	50-400	.086

PLC (10^9^)	175	115-220	74	60-190	.037*

BU (mmol/L)	5	4-10	14	5-26	.017*

K (mmol/L)	4	4-5	4	4-5	.755

Creatinine (mg/dl)	0.80	0.60-1.20	2.80	.00-4.00	.0001*

*IQR***- **Inter quartile range, ^1 ^Based on Mann-Whitney U test

*Significant

In the univariate analysis, patients with complications had lower Hb, PCV, and platelet count with higher WBC counts, blood urea, and creatinine levels compared to those without complications. We used Mann-Whitney *U *test due to non-normal distribution of data.

Those clinical features and laboratory measures were then entered into a logistic regression model to assess the predictor values of each variable.

Table 3 shows the results of logistic regression model.

**Table 3:** Variables included in the final model that predicts leptospirosis complications

Clinical feature	***p***	Odds ratio	95% confidence interval
Oliguria	.049*	4.140	1.003 to 17.261

Jaundice	.033*	5.673	1.149 to 28.003

Arrhythmias	.050*	5.774	1.001 to 34.692

*Significant

In the backward stepwise elimination process only three variables were retained in the model. Oliguria, jaundice and arrhythmias on admission were shown as predictors of acute renal failure or myocarditis.

## Discussion

Clinical presentation of leptospirosis can be varied and non specific, hence the recognition of red flags of complications is of paramount importance. The aim of our study was to determine such red flags and to formulate a risk predictor model. Of the many clinical and laboratory predictors of mortality which have been described, we assessed nine clinical and eight laboratory variables in our study. Furthermore many studies have been conducted to determine the predictors of mortality, but studies conducted to determine the predictors of complications were not found in Sri Lankan literature [18].

Out of the 17 variables assessed, only oliguria, jaundice and arrhythmias were predictors of leptospirosis complications. Although previous studies have shown that 70% of leptospirosis patients were having arrhythmias, in our study it was only 12.9%. In another study acute renal failure was found in 40% of patients which is a higher value compared to ours. In the same study it was shown that oliguria is a sensitive sign of severe leptospirosis. This finding is somewhat similar to the finding of ours. Why the clinical severity of leptospirosis varies so much is so far unexplained but difference in serovars could be a good reason. Although univariate analysis showed a significant difference for most of the laboratory variables, none of them were significant as predictors in multivariate analysis. Similarly, oliguria and jaundice showed a significant difference as predictors in multivariate analysis despite having no significant difference in univariate analysis. This finding could be due to the confounding effects of the variables. All three variables included in the predictor model were strong predictors and are included in surveillance case definition of leptospirosis in Sri Lanka as well as other countries.

Although jaundice was found to be a predictor of complications in our study, a study done in Turkey [19] has shown that majority of their patients had anicteric leptospirosis which could also lead to severe disease. Many previous studies have shown leucocytosis, thrombocytopenia and blood urea as early predictors [20]. Interestingly, none of the laboratory measures were shown to have any value as early predictors of renal or cardiac complications in our study. This is a valuable finding as most patients present to rural or district hospitals which lack laboratory facilities, therefore predicting their complications and need for transfer could be decided using the three clinical criteria alone.

Although many previous studies have shown pulmonary involvement with haemoptysis as a major complication [14], it was not found in our study. Possible reason for this could be a different pathogenic serovar circulating in the area, but this could not be confirmed as we didn't analyze for serotypes.

Thrombocytopenia was a common finding in our patients. Despite this bleeding was uncommon and unlike in other studies it did not predict the development of myocarditis or acute renal failure. Similarly leucocytosis was also a common finding and it is a valuable investigation in differentiating leptospirosis from dengue fever which is deadlier than leptospirosis. It was also not proven to be effective as a predictor of complications.

As seen with other similar studies [21] acute renal failure was a major complication in our patients. Oliguria has shown to predict acute renal failure but not BU, K, and creatinine. It may be that serum biochemical markers lag behind the clinical presentation of acute renal failure in leptospirosis but the exact mechanism is unknown.

As oliguria, jaundice and arrhythmia point towards involvement of three distinct organ systems, it is possible that all three clinical features herald multi organ involvement in severe disease with different organs involved in different severity.

Many outcome prediction scores have been developed for the critically ill patients e.g. APACHE 11, but none of them have been validated for leptospirosis. According to our study, presence of the three clinical variables oliguria, jaundice and arrhythmias predict acute renal failure and myocarditis hence can be utilized to decide which patient should receive intensive/high dependency care.

### Limitations of the study

Present study was carried out within the limited resources for diagnosis of leptospirosis as shown previously. Due to this lack of point of care diagnostic facilities, there were several limitations to our study. Although none of the laboratory confirmed cases died, three patients suspected of having leptospirosis died before serology could be performed, and were not included in the study, which could have introduced a selection bias in the sample. The cases included were based on a single sample MAT titre, which is not the standard test for confirmation. Further, it has been shown that development of antibody might take more than seven days in some patients. These could have missed out some of the patients with leptospirosis. Previous studies have shown that complications of leptospirosis depend on infecting serovars, which in this study we were unable to study due to resource limitations. Although all patients were studied as a single sample, complications and predictors could be varied for different serovars. Duration from the onset of the illness to the admission was varied which could have had some confounding effect on the investigations done on admission. This could be a reason why laboratory measures were poor in predicting the complications. Due to the lack of facilities we couldn't confirm myocarditis histologically and it was done with clinical, electrocardiographical and echocardiographical evidence. But histological confirmation would not change the management of a leptospirosis patient with myocarditis.

## Conclusions

This study shows that out of 17 variables, only oliguria, jaundice and arrhythmia are strong predictors of development of acute renal failure or myocarditis and none of the laboratory variables could predict these complications in patients with leptospirosis presented to Teaching Hospital of Kandy, Sri Lanka.

## Competing interests

The authors declare that they have no competing interests.

## Authors' contributions

DLBD was involved in designing, acquisition of data and drafting the manuscript of the study. HW conceived the study, reviewed the manuscript and approved the final manuscript. SBA was involved in statistical analysis, interpretation of data and review of the manuscript. DN was involved in the acquisition of data, and designing the study. AN was engaged in designing the study, and interpretation of data. CNR was involved in data acquisition, and review of the manuscript. All authors approved the final manuscript.

## Authors' information

DLBD: Is the corresponding author and is a registrar in Medicine attached to wards 33 and 35 of the Teaching Hospital Kandy. He has an MBBS degree and is enrolled in the MD Medicine Training Program in the Postgraduate Institute of Medicine, Colombo.

HW is the Consultant Physician in charge of the Medical Unit, wards 33 and 35 in the Teaching Hospital Kandy. He has obtained MD, FRCP and FCCP and is a member of the College of Physicians Sri Lanka.

DN is an MBBS graduate working as an Intern House Officer in the Teaching Hospital Kandy.

AN is an MBBS graduate working as an Intern House Officer in the Teaching Hospital Kandy.

CNR is an MBBS graduate working as an Intern House Officer in the Teaching Hospital Kandy.

SBA is a Lecturer in Community Medicine in Faculty of Medicine and Allied Sciences, University of Rajarata, Sri Lanka and a Visiting Assistant Professor, Division of Infectious Diseases, Department of Medicine, UCSD School of Medicine, USA. He has an MD in Community Medicine and MPH (Bio-security).

## Pre-publication history

The pre-publication history for this paper can be accessed here:

http://www.biomedcentral.com/1471-2334/12/4/prepub
